# Vehicle images dataset for make and model recognition

**DOI:** 10.1016/j.dib.2022.108107

**Published:** 2022-03-29

**Authors:** Mohsin Ali, Muhammad Atif Tahir, Muhammad Nouman Durrani

**Affiliations:** School of Computer Science, National University of Computer and Emerging Sciences, Karachi Campus, Pakistan

**Keywords:** Image data-set, Vehicle model recognition deep learning, Machine learning

## Abstract

Vehicle make and model recognition plays an important role in monitoring traffic in a vehicle surveillance system. Identifying vehicle make and model is a challenging task due to intraclass variation, view-point variation, and different illumination conditions (Hassan et al., 2021). In this domain, many datasets regarding car make and model e.g. Stanford Car (Krause et al., 2013), VMMRdB (Tafazzoli et al., 2017, Yang et al., 2015), have already been experimented with by different researchers. However, most of the images in these datasets are high-quality images with no illumination conditions. Further, these images are collected through web crawling or image scraping. This enabled the researchers to achieve good results using deep learning models (Luo et al., 2015). In this article, we have presented an image dataset of 3847 images, designed from high-resolution (1920 1080) videos collected from camera units installed on a highway at different viewpoints with variable frame rates. This helped in collecting images demonstrating a real-world scenario and made this dataset more challenging. Due to consideration of different viewpoints and illumination effects, the dataset will help researchers to evaluate their machine learning models on realworld data (Manzoor et al., 2019).

## Specifications Table

**Table:** 

Subject	Computer Vision
Specific subject area	Vehicle Model Recognition
Type of data	Images
How data were acquired	The video was recorded with a high frame rate camera with 8 mm autofocus and F2.0 aperture. The camera was mounted at the back of the vehicle using a mounting stand.
Data format	Raw Data
Parameters for data collection	Data was collected between 10 am to 4 pm outdoor in an uncontrolled environment
Description of data collection	After collection of videos in hig resolution in an uncontrolled environment, frames were extracted from videos which contain vehicles. Later appropriate vehicles were cropped out of the frames and manually labelled according to vehicle make and model.
Data source location	Institution: National University of Computer and Emerging Sciences City/Town/Region: Karachi Country: Pakistan
Data accessibility	Repository name: Mendeley Data Data identification number: 10.17632/hj3vvx5946.1 Direct URL to data: https://data.mendeley.com/datasets/hj3vvx5946/1 Data is also attached with the manuscript

## Value of the Data

• The images in the dataset are collected at different time frames with different angles. The dataset contains images with variety of lighting conditions and angles which help in demonstrating real-world scenarios.
• As the dataset contains images demonstrating real-world scenarios. This will help re- searchers to evaluate their existing deep learning models, trained on different datasets.
• This dataset will help researchers already working on the vehicle make and model recog- nition systems to train and test their model performance on the real world data.
• In this dataset, all vehicles images are divided into Train and Test splits. Further, these images are then annotated into 48 different classes respectively according to their vehicle make and model.

## 1 Data Description

**Fig. 1:**
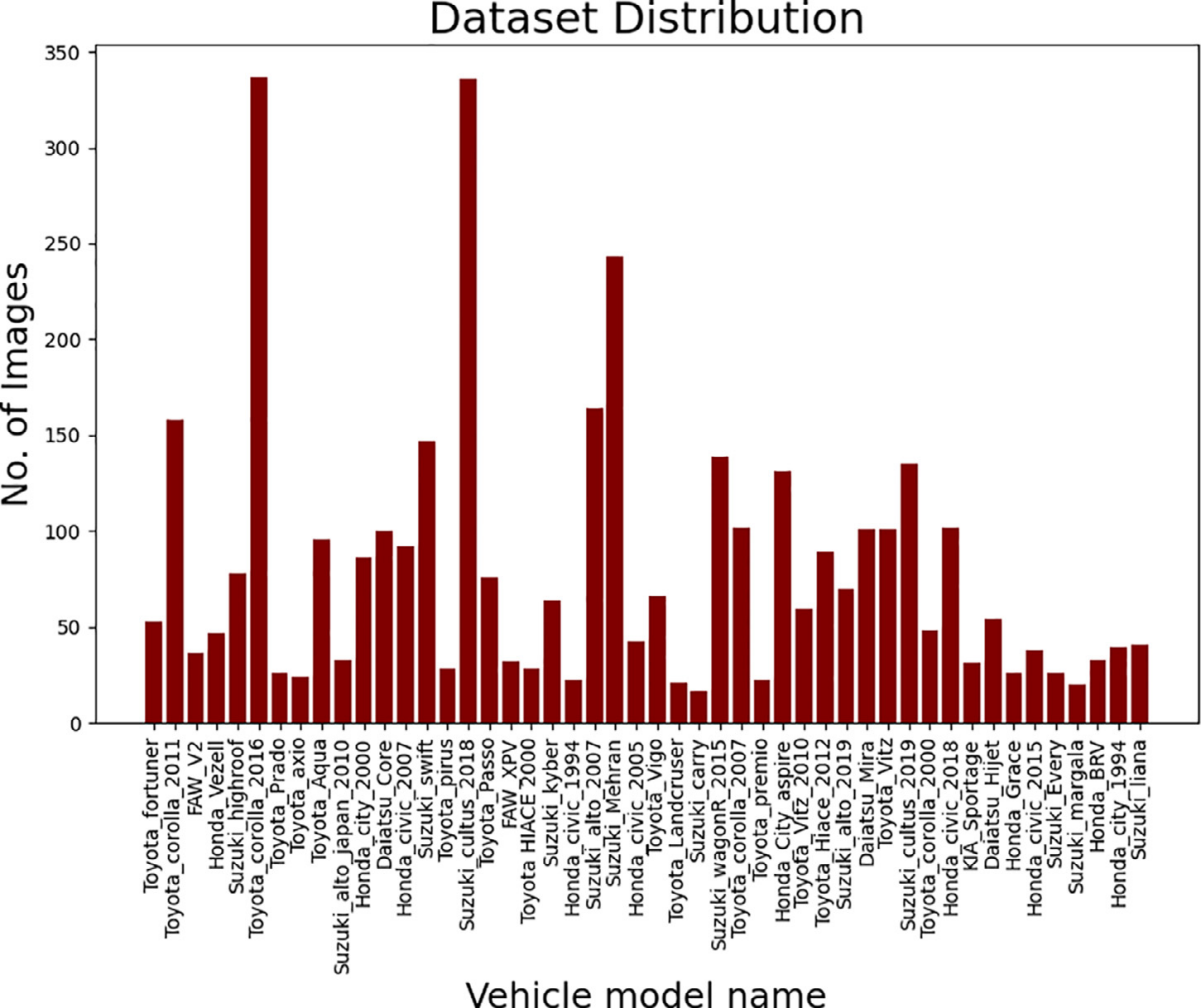
Distribution of total images in different classes.
• Fig. 2 show sample images randomly picked from the dataset.

• The dataset [8] consists of 3847 images of different vehicles make and model.
• The dataset has 48 different classes of vehicle models which are annotated in 48 different folders. Each folder is named with its respective vehicle make and model name.
• Fig. 1 visually describes the distribution of the dataset according to its classes.

**Fig. 2:**
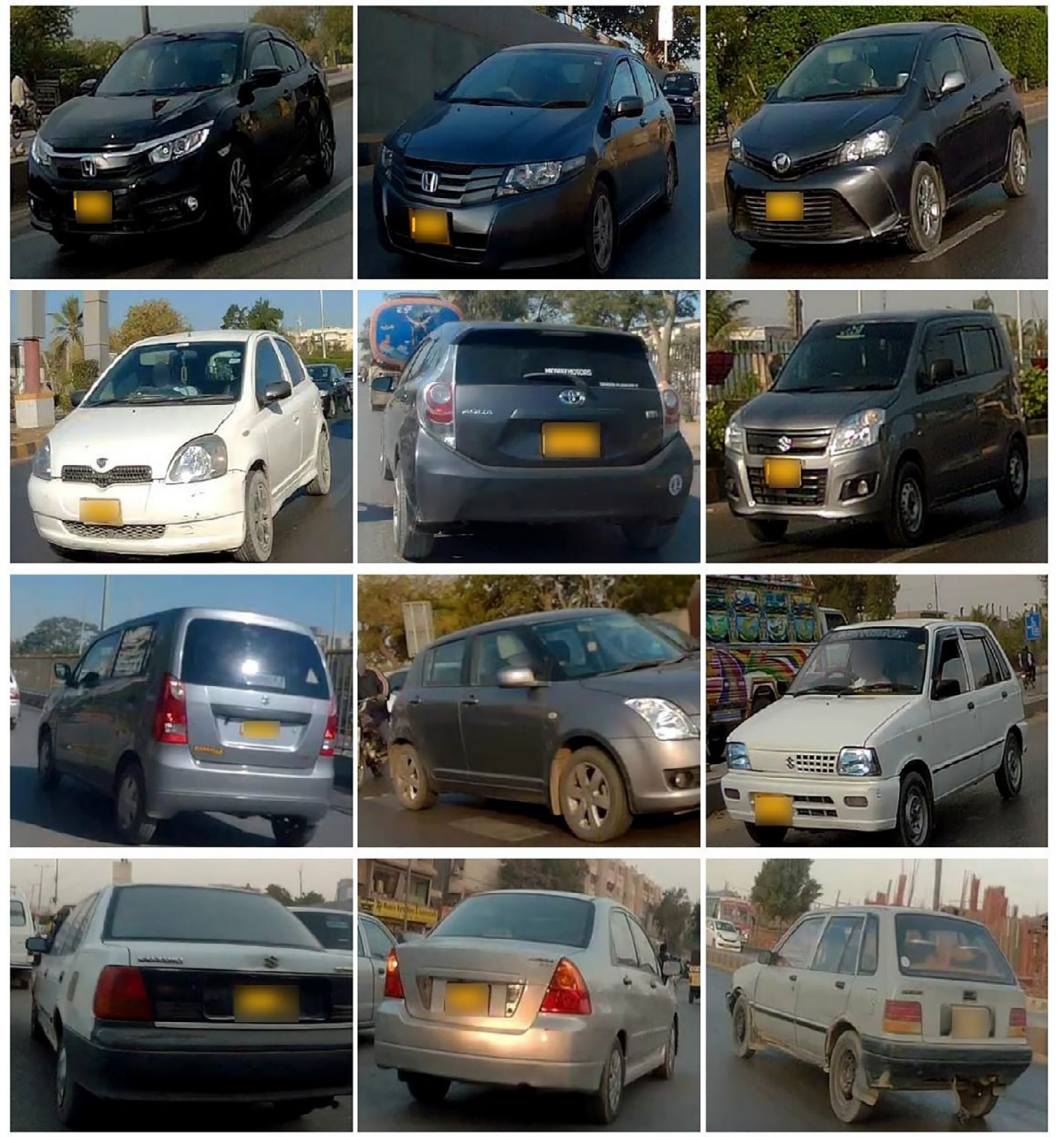
Sample image in the dataset.
• Table 1 describe the features in comparsion of our [8] with other similar datasets.

**Table 1:** Comparison with other datasets.

S No.	Dataset	Description
1	AOLP [7]	This dataset is mainly focuses on vehicle licence plate. This dataset mostly contain images of Chinese models.
2	CompCar [4]	In this dataset most of the images are extracted CCTV cameras. Further, Most of the car images in this dataset are of Chinese models.
3	Stanford Cars Dataset [2]	This dataset mainly focus on vehicle make and model. Most of the images in this dataset are scrap from the Internet, some images are also extracted from car sales website and movies
4	VMMRdb [3]	It is one of the largest dataset for vehicle make and model recognition. It contain about 9170 classes. But images in this dataset are highly imbalanced.
5	*Our*[8]	The images in this dataset is collected using high resolution camera. Images of the vehicle is taken from different camera angles and lightning conditions to simulate real-world conditions.
• Table 2 show the initial results obtain on pre-trained deep learning models.

**Table 2:** Initial results using pre-trained deep learning models.

S No.	Model	Accuracy
1	ResNet 50	67.13%
2	ResNet152	69.24%
3	MobileNet	73.54%
4	VGG16	74.32%

## 2 Experimental Design, Materials and Methods

### 2.1 Data collection

In the first step, videos were collected using a standard vehicle high-resolution video camera at a variable frame rate, between 10 am to 7 pm. The videos were recorded on the main national highway, in front of the university, and for consent with the individuals’ signboard of video recording disclaimer was installed.

### 2.2 Data processing

In this section, the data processing performed on the collected videos has been discussed. As this earlier, the videos were collected using a standard mounted video camera. These videos were split into frames with an interval of 1 s. In the next step, similar frames were cleaned manually. Further, In order to maintain the privacy of the individuals, most of the background was removed and vehicle images were only extracted, using manual cropping. Further, these images were annotated into 48 different folders. Each folder was named with the make and model of the vehicle. In order to secure the identity of the vehicle owner, the number plate characters were blurred. Moreover, to maintain privacy in the dataset faces of all individuals in the dataset were blurred manually.

**Fig. 3:**
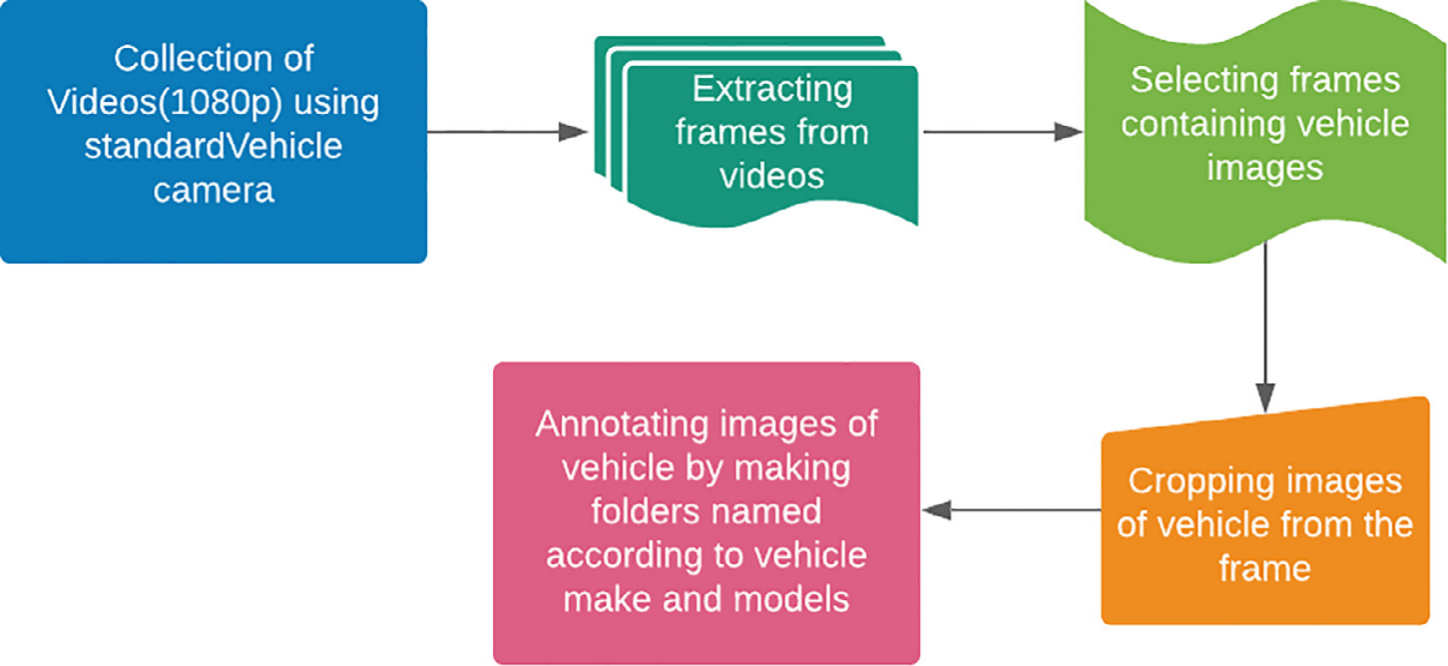
Process for dataset creation.

After annotation of these images, the data was split into the 20% Testing part and 80% Training so that different machine learining models can be evaluated using this data. Dataset creation process is shown in Fig. 3.

### 2.3 Experimental setup and results

To obtain the initial results of the deep learning model on our dataset we used a high-performance machine having 8GB of GPU RAM (Nvidia p6000). Further, we used TensorFlow and Keras as deep learning libraries for training models. We conducted the experiment on the same test, train split as it is given by the dataset, for validation data we further split training data to 90% training and 10% validation. We load all the models with the ImageNet [9] weights and used the transfer learning technique which reduce the training time of the models and train the models on 50 epochs initially [1], [6]. We used accuracy as a performance parameter and initial results are given below [5]. In the future, these results can be improved by using data balancing techniques, ensemble learning techniques, etc.

## Ethics Statement

All authors ensure this article “Vehicle images dataset for the vehicle make and model recognition” fulfil the following ethics requirements:

• This dataset is not been published anywhere else.
• Nor this article is consider for publication anywhere else.
• None Experiments was conducted on human or animals.
• Approval was obtained by research ethics committee to conduct this study.
• To make sure individuals are aware that they are being recorded data was only collected from the front of the university having disclaimer of video recording installed.
• Further, to secure the personal information, license plates and faces of the individuals were blurred.

## CRediT authorship contribution statement

**Mohsin Ali:** Data curation, Writing – original draft. **Muhammad Atif Tahir:** Writing – review & editing. **Muhammad Nouman Durrani:** Data curation.

## Declaration of Competing Interest

The authors declare that they have no known competing financial interests or personal relationships which have, or could be perceived to have, influenced the work reported in this article.

## Data Availability

Vehicle images dataset for make and model recognition (Reference data) (Mendeley Data).

## Appendix Supplementary Materials

Image, application 1
